# Characterising intra-articular corticosteroid injections use in UK primary care: a retrospective cohort study between 2005 and 2020

**DOI:** 10.1093/rap/rkag085

**Published:** 2026-07-17

**Authors:** Ffion Samuels, Albert Prats-Uribe, Anna Saura Lazaro, Samuel Hawley, Gulraj S Matharu, Antonella Delmestri, Andrew Judge, Michael R Whitehouse, Daniel Prieto-Alhambra

**Affiliations:** Health Data Sciences Section, Nuffield Department of Orthopaedics, Rheumatology and Musculoskeletal Sciences, Botnar Research Centre, Oxford, UK; Health Data Sciences Section, Nuffield Department of Orthopaedics, Rheumatology and Musculoskeletal Sciences, Botnar Research Centre, Oxford, UK; Health Data Sciences Section, Nuffield Department of Orthopaedics, Rheumatology and Musculoskeletal Sciences, Botnar Research Centre, Oxford, UK; Musculoskeletal Research Unit, Translational Health Sciences, Bristol Medical School, University of Bristol, Bristol, UK; Musculoskeletal Research Unit, Translational Health Sciences, Bristol Medical School, University of Bristol, Bristol, UK; Health Data Sciences Section, Nuffield Department of Orthopaedics, Rheumatology and Musculoskeletal Sciences, Botnar Research Centre, Oxford, UK; Musculoskeletal Research Unit, Translational Health Sciences, Bristol Medical School, University of Bristol, Bristol, UK; National Institute for Health Research Bristol Biomedical Research Centre, University Hospitals Bristol and Weston NHS Foundation Trust and University of Bristol, Bristol, UK; Musculoskeletal Research Unit, Translational Health Sciences, Bristol Medical School, University of Bristol, Bristol, UK; National Institute for Health Research Bristol Biomedical Research Centre, University Hospitals Bristol and Weston NHS Foundation Trust and University of Bristol, Bristol, UK; Health Data Sciences Section, Nuffield Department of Orthopaedics, Rheumatology and Musculoskeletal Sciences, Botnar Research Centre, Oxford, UK

**Keywords:** osteoarthritis, corticosteroid, ankle, foot, elbow, hand, hip, knee, shoulder, wrist

## Abstract

**Objectives:**

To characterise the use of intra-articular corticosteroid injections (IACIs) in a large patient cohort with OA in UK primary care from 2005 to 2020. We sought to evaluate IACI practices, identify patterns of administration across different joints, assess regional variation and examine potential demographic factors influencing IACI utilisation for managing OA.

**Methods:**

This retrospective cohort study used data from the UK Clinical Practice Research Datalink GOLD, a database of primary care health records. Data from other healthcare settings were not included. Patients ≥20 years of age with a first diagnosis of OA between 2005 and 2019 were included. OA and IACIs were identified through Read and Gemscript codes, respectively.

**Results:**

Of 220 125 patients with OA, 11% received at least one IACI. The anatomical location was only specified in 56% of IACI records. The highest proportion of injections occurred in patients with multiple joints diagnosed with OA (19.0%), followed by the shoulder (14.9%) and knee (12.5%). The median time from OA diagnosis to first injection was 0.9 years and was shortest for the shoulder (0.3 years) and longest for the elbow (2.1 years). We observed substantial regional variation, with incidence rates ranging from 1.2 injections per 100 patient-years in the South East Coast to 3.8 in Yorkshire and the Humber.

**Conclusion:**

This study observed notable variation in the median time from OA diagnosis to injection, across different joints, with substantial heterogeneity in the incidence of IACIs across UK regions.

Key messages

- The joints most commonly treated with IACIs differed from those most commonly affected by OA.
- Notable variation existed in the median time from OA diagnosis to the first injection across joint sites.
- Regional variation in the incidence of IACIs in the UK was substantial.

## Introduction

OA is the most common form of arthritis and the most prevalent musculoskeletal condition worldwide, with >350 000 cases diagnosed annually in the UK [1, 2]. Hip and knee OA are leading causes of global disability and chronic pain. Additionally, OA is associated with substantial costs for healthcare systems and society [3–5].

The prevalence of OA is highest in females and increases between the ages of 45 and 75 years [6]. Due to ageing populations and the increasing incidence of obesity, it is predicted that demand for OA treatment will increase [7]. By 2050, an estimated 130 million people will be affected by OA globally, of whom 40 million (31%) are projected to be severely disabled by the disease [8].

The first UK National Institute for Health and Care Excellence (NICE) guideline for the management of OA, CG59, was published in 2008, recommending that intra-articular corticosteroid injections (IACIs) should be considered as an adjunct to core treatments for the relief of moderate to severe pain [9]. This recommendation was retained in the updated guideline, CG177, published in 2014, which noted that the evidence base demonstrated that IACIs provide short-term pain reduction of 1–4 weeks, with less marked effects on function [10]. The most recent NICE guideline, NG226, was published in 2022, which was outside our study period [11]. According to the British National Formulary (BNF), IACIs can be repeated at intervals of 7–35 days, but each joint should not usually be treated more than four times in 1 year [12].

NICE acknowledges that there is a lack of consistent evidence for the use of IACIs, especially for joints other than the knee and therefore recommends further research [11]. A recent review further highlighted the absence of published data on the frequency of administration of IACIs for OA, noting that much of the current guidance is based on expert opinion [13]. It is widely recognised that data on patterns of use of IACIs after treatment initiation and the factors impacting these patterns are inadequate. Significant knowledge gaps also exist in our understanding of gender-based differences in treatment patterns and potential regional variations in IACI administration across the UK healthcare system, given that these factors may influence patient access to treatment. Thus, further research into the current practice and patterns of IACI use is required to inform and enable future evidence-based OA guidance on appropriateness of use, dosing intervals and treatment frequency.

This study aimed to characterise the use of IACIs in a large, representative cohort of patients with OA in UK primary care over a 15-year period, using routinely collected observational primary care data.

## Methods

### Study design, setting and population

We conducted a cohort study using routinely collected UK observational primary care data, as one component of the wider RecUrrent Intra-articular Corticosteroid injections in Osteoarthritis (RUbICOn; NIHR129011) study. The aims of RUbICOn included evaluating patterns of use, long-term effects and safety of IACIs, exploring patient and clinician perspectives and identifying research priorities and feasibility considerations for future studies.

Under the UK’s National Health Service (NHS), primary care is provided free at the point of delivery by general practitioners (GPs), with almost the entirety of the population registered to a local GP practice [14]. Patients with OA are typically managed in primary care and treated without surgery.

We included patients ≥20 years of age with a first diagnosis of OA in the ankle/foot, elbow, hand, hip, knee, shoulder or wrist in the primary care sector between 1 January 2005 and 31 December 2019. We excluded patients with a body mass index <15 kg/m^2^ at the time of their first OA diagnosis and patients with a referral for orthopaedic surgery before their first OA diagnosis date.

### Data source

Data were extracted from the Clinical Practice Research Datalink (CPRD) GOLD for the period 1 January 2005 to 28 August 2020. CPRD GOLD is an electronic database of UK pseudo-anonymised primary care health records administered by the Medicines and Healthcare products Regulatory Agency [14]. At the time of data extraction (September 2020), CPRD GOLD cumulatively contained data covering ≈19 million people from >900 GP practices spread across the country. The database has been shown to be representative of the UK in terms of age, sex and ethnicity [14]. Symptoms, diagnoses, operations, procedures and other health-related information are recorded into CPRD GOLD and mapped to Read codes. Prescriptions data are mapped to Dictionary of Medicines and Devices (dm + d) and Gemscript codes. Extensive work validates the coding in CPRD GOLD [15]. Only patients whose data quality was flagged as ‘acceptable’ for clinical research were included from GP practices that had uploaded at least 1 year of ‘up-to-standard’ data. CPRD GOLD assigns the ‘up-to-standard’ quality flag based on data quality checks at the GP practice level [14].

### Study variables and cohort definitions

We included the following variables: date of first OA diagnosis, anatomical site of OA and date of IACI administration. OA and IACI were identified through Read and Gemscript codes, respectively, as described in Supplementary Tables S1 and S2. Additional variables collected included age at first OA diagnosis (calculated from the year of birth), sex (as recorded in GP data) and geographical region of residence.

We created separate cohorts for each OA anatomical site studied: ankle/foot, elbow, hand, hip, knee, shoulder and wrist. Where patients had more than one coded OA anatomical site, this was considered OA affecting multiple joints. We treated each unique CPRD GOLD patient identifier (patid) as a different patient, as this has previously been shown to be a valid approach for handling the small number of patient transfers between CPRD GOLD–registered GP practices [16].

### Statistical analysis

We extracted counts of OA per anatomical site and described the distribution of OA sites among identified patients using proportions. We reported the sex distribution, the mean and s.d. of age at first OA diagnosis, both overall and by OA site.

For IACIs, we extracted counts of IACIs and the number and proportion of OA patients who received at least one IACI, hereafter referred to as injected patients. We estimated the mean number of IACIs per OA patient by dividing the number of injected patients by the total number of OA patients. We calculated the mean number of IACIs per injected patient, both overall and by OA site, by dividing the number of injected patients by the total number of OA patients who received at least one IACI. Additionally, we estimated the mean and median time since OA diagnosis to injection.

To analyse the IACI utilisation patterns, we calculated 1- to 5-year incidence as the number of injections per person time, starting the follow-up time at OA diagnosis and censoring at death. We also calculated the prevalence of first injection or any injection after OA diagnosis by anatomical site. Incidence of first injection after OA diagnosis was calculated by calendar year, with a burn-in period in 2005–2006, as the number of patient-years was too low to obtain stable estimates. Incidence per 100 person-years of injections was also calculated by age group (<30, 30–39, 40–49, 50–59, 60–69, 70–79, 80–89, 90+) and sex, stratified by OA anatomical site. Finally, we computed incidence rates of injection by anatomical joint and UK region, and yearly incidence rates of injection by anatomical joint.

### Ethics

This study was approved via the CPRD Research Data Governance process (ref: 20_067RA). No informed consent was required, as this study used pseudo-anonymised, routinely collected data.

## Results

A total of 220 125 patients in the primary care sector were diagnosed with OA within the study period (Table 1). Of these, 58.8% were women. Anatomical location was only specified in 56% of IACI injection records, preventing definitive identification of the injected joint site(s) in the remainder. The elbow was the only OA anatomical site where men were more affected than women, with only 30.0% of cases occurring in women. In contrast, hand OA had the highest proportion of female patients (72.4%). The mean age at first OA diagnosis was 65.4 years (s.d. 12.4). Patients with shoulder OA had the highest mean age at first OA diagnosis (67.9 years), whereas the youngest mean age at diagnosis was observed among individuals with ankle or foot OA (61.2 years). The most frequent OA anatomical sites were the knee [*n* = 106 890 (48.6%)], hip [*n* = 50 970 (23.2%)] and hand [*n* = 27 820 (12.6%)]. There were 17 230 (7.8%) patients with OA at more than one site.

**Table 1:** Number of diagnosed patients, percentage of females and mean age at diagnosis by OA site between 2005 and 2019.

OA site	*n* (%)	Female, %	Age at first OA diagnosis, years, mean
Knee	106 890 (48.6)	56.0	65.6
Hip	50 970 (23.2)	57.7	66.7
Hand	27 820 (12.6)	72.4	62.8
Ankle or foot	8550 (3.9)	52.5	61.2
Shoulder	5490 (2.5)	52.7	67.9
Wrist	2351 (1.1)	59.4	66.0
Elbow	824 (0.4)	30.0	61.8
Multiple joints	17 230 (7.8)	63.7	66.6
Total	220 125	58.8	65.4

A total of 23 899 (10.9%) OA patients received at least one IACI on or after the date of their first OA diagnosis. OA affecting multiple joints had the highest proportion of OA patients receiving an IACI (19.0%), followed by shoulder OA (14.9%) and knee OA (12.5%) (Table 2). Supplementary Table S3 shows the number of patients with each combination of joint OA. Among OA patients, the average number of IACIs was highest in those with OA in multiple joints (0.43), followed by the knee (0.27) and shoulder (0.25). Among injected patients, excluding patients with OA in multiple joints, the knee (42.9%) and hand (38.4%) were the anatomical sites with the highest proportion of patients injected multiple times, with a mean of 2 injections per patient for both regions. Injected patients with OA in multiple joints had the highest mean number of injections per injected patient (2.3), followed by those with knee OA (2.2) and hand OA (1.9).

**Table 2:** Proportion of OA patients receiving injections and the proportion of patients receiving more than one injection by OA site.

OA site	OA patients, *n*	Injections, *n*	Injected patients, *n*	OA patients injected, %	Patients with >1 injection, %	Mean injections per OA patient, *n*	Mean injections per injected OA patient, *n*
Knee	106 890	28 938	13 391	12.5	42.9	0.3	2.2
Hip	50 970	5793	3422	6.7	33.3	0.1	1.7
Hand	27 820	4054	2085	7.5	38.4	0.2	1.9
Ankle or foot	8550	1226	662	7.7	37.0	0.1	1.9
Shoulder	5490	1362	815	14.9	34.8	0.3	1.7
Wrist	2351	355	205	8.7	30.2	0.2	1.7
Elbow	824	78	53	6.4	26.4	0.1	1.5
Multiple joints	17 230	7452	3266	19.0	45.6	0.4	2.3

The time from OA diagnosis to injection varied depending on the anatomical site. The shortest median time from OA diagnosis to the first injection was observed for the shoulder (median 0.3 years) and the knee (median 0.7 years), while the longest times were observed for the elbow (median 2.1 years), the hand (median 1.5 years) and the hip (median 1.5 years) (Table 3).

**Table 3:** Time from diagnosis to injection for the first to the sixth injection by OA site.

OA site	Injection 1			Injection 2			Injection 3			Injection 4			Injection 5			Injection 6		
	*n*	Mean	Median	*n*	Mean	Median	*n*	Mean	Median	*n*	Mean	Median	*n*	Mean	Median	*n*	Mean	Median
Knee	13 391	2.0	0.7	5747	2.7	1.6	3077	3.4	2.5	1876	3.8	3.0	1239	4.2	3.5	857	4.5	3.9
Hip	3422	2.5	1.5	1141	3.5	2.8	510	4.2	3.4	267	5.1	4.5	138	5.4	4.6	82	5.7	5.3
Hand	2085	2.6	1.5	801	3.4	2.4	396	3.8	2.9	251	4.2	3.3	152	4.7	3.9	107	5.3	5.1
Ankle or foot	662	2.3	1.2	245	3.2	2.2	117	4.0	3.3	63	4.3	3.0	44	5.0	4.4	31	5.1	3.8
Shoulder	815	1.4	0.3	284	2.2	1.3	118	3.2	2.4	57	4.0	2.8	32	4.5	4.0	22	4.9	3.8
Wrist	205	2.2	1.0	62	2.7	2.1	30	3.4	2.8	17	3.7	2.5	13	5.1	3.0	6	2.4	2.4
Elbow	53	2.7	2.1	14	3.7	3.5	<5	2.4	2.1	<5	4.6	4.6	<5	4.1	4.1	<5	4.3	4.3
Multiple joints	3266	2.4	1.2	1489	3.2	2.2	811	3.9	3.0	490	4.4	3.6	316	4.9	4.1	226	5.1	4.6

Analysis of IACI utilisation patterns revealed distinct variations across different OA anatomical sites (Table 4). Cumulative IACI uptake over time after OA diagnosis was higher in those with multiple joint OA, with a cumulative incidence of 9.0% at 1 year, progressively increasing to 15.7% by year 5 post-diagnosis (Table 4). Those with shoulder and knee OA had a 5-year cumulative incidence of 13.6% and 10.8%, respectively. In contrast, elbow and hip OA had the lowest IACI utilisation, with 5-year cumulative incidence rates of 5.2% and 5.6%, respectively. The highest prevalence of IACI use was observed in shoulder OA, with a 5-year prevalence of 10.4%, followed by multiple joint OA (9.3%) and knee OA (6.1%) (Table 4). The lowest prevalence of IACI use was observed in hip OA, with a 5-year prevalence rate of 4.1%.

**Table 4:** Yearly prevalence and cumulative incidence of injections (up to 1 per person-year) after diagnosis by OA site.

Prevalence	Knee, %	Hip, %	Hand, %	Ankle or foot, %	Shoulder, %	Wrist, %	Elbow, %	Multiple joints, %
1 year	3.1	1.7	1.4	1.8	6.2	2.3	1.6	4.2
2 year	4.3	2.5	2.4	2.5	8.0	3.2	2.8	6.2
3 year	5.0	3.2	3.2	3.2	8.9	4.0	3.3	7.4
4 year	5.7	3.7	3.8	3.8	9.6	4.8	3.8	8.5
5 year	6.1	4.1	4.2	4.2	10.4	5.2	4.3	9.3
**Incidence**								
1 year	6.9	2.8	3.1	3.6	10.1	4.5	2.1	9.0
2 year	8.4	3.9	4.3	4.6	11.8	5.6	3.2	11.5
3 year	9.4	4.6	5.0	5.4	12.6	6.4	4.1	13.2
4 year	10.2	5.1	5.6	6.1	13.2	7.0	4.7	14.6
5 year	10.8	5.6	6.1	6.6	13.6	7.3	5.2	15.7

**Figure 1:**
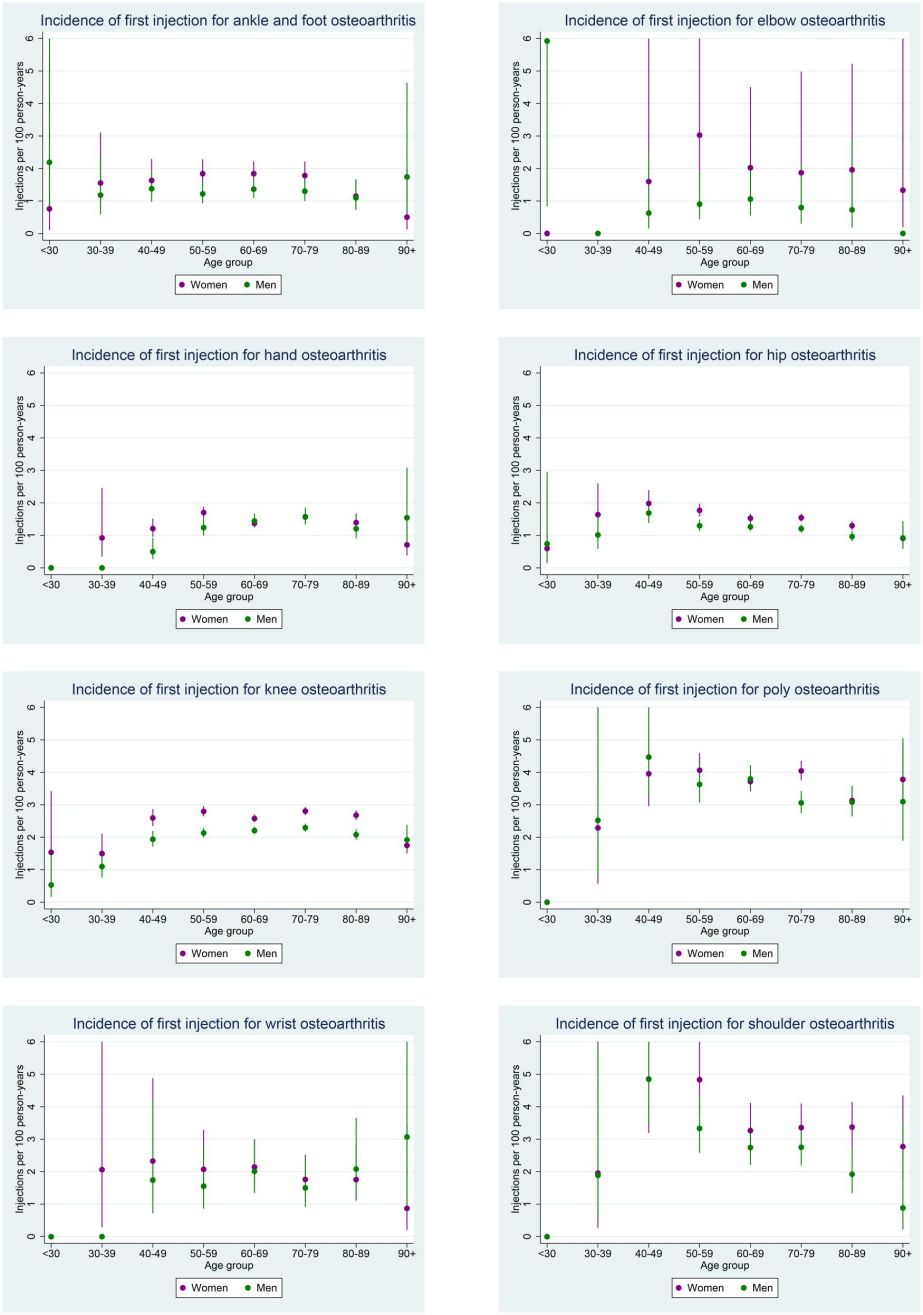
Incidence of first injection after OA diagnosis, by OA site, age group and sex. The incidence of first injection per 100 person-years is represented by a dot, with the line signifying the 95% CI. Purple denotes women and green denotes men

The incidence of a first injection for most OA anatomical sites increases with age until ≈50 years, remains relatively stable until 80 years and then begins to decline (Fig. 1). An exception is hip OA, where the peak appears to occur in the 40- to 49-year age group. Estimates for wrist and elbow OA are highly uncertain, making it difficult to discern any clear trends. Across all OA anatomical sites, incidence rates of IACI use were consistently higher in women than men, with notable differences for knee OA (2.7 *vs* 2.2 per 100 person-years) and hip OA (1.5 *vs* 1.2 per 100 person-years).

Additionally, the use of IACI varied greatly by UK geographical region (Fig. 2). For all OA anatomical sites combined, the UK region with the highest incidence of IACIs was Yorkshire and the Humber, with a rate of 3.8 (95% CI 3.5, 4.1) injections per 100 patient-years with OA, followed by the East Midlands with 3.4 (95% CI 3.1, 4.7) and Northern Ireland with 3.4 (95% CI 3.2, 3.5). The regions with the lowest incidence of IACIs were the South East Coast [1.2 (95% CI 1.1, 1.3)] and the South West [1.4 (95% CI 1.3,1.5)]. This ranking was mostly consistent when looking at each OA anatomical site separately.

**Figure 2:**
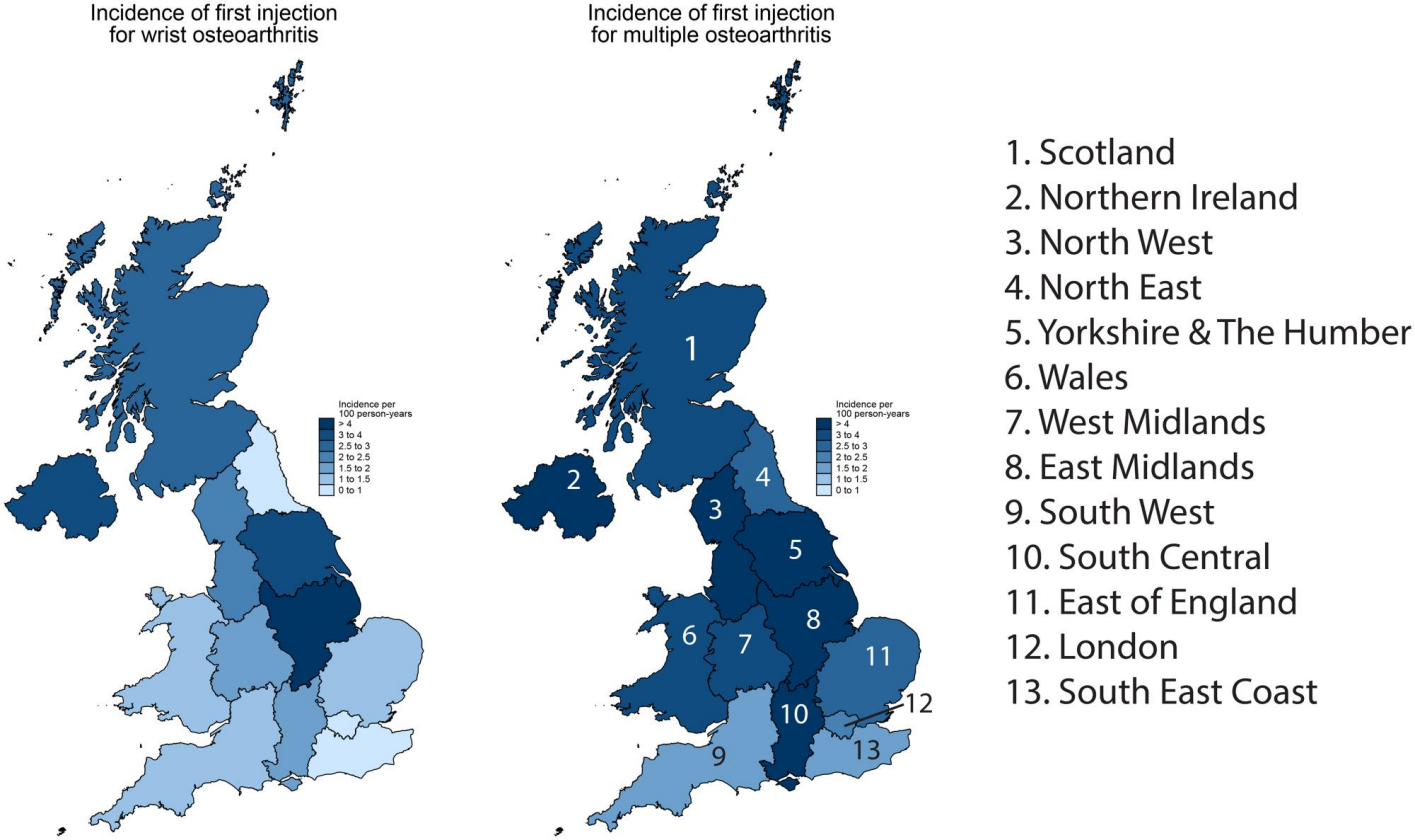
Continued.

**Figure 2:**
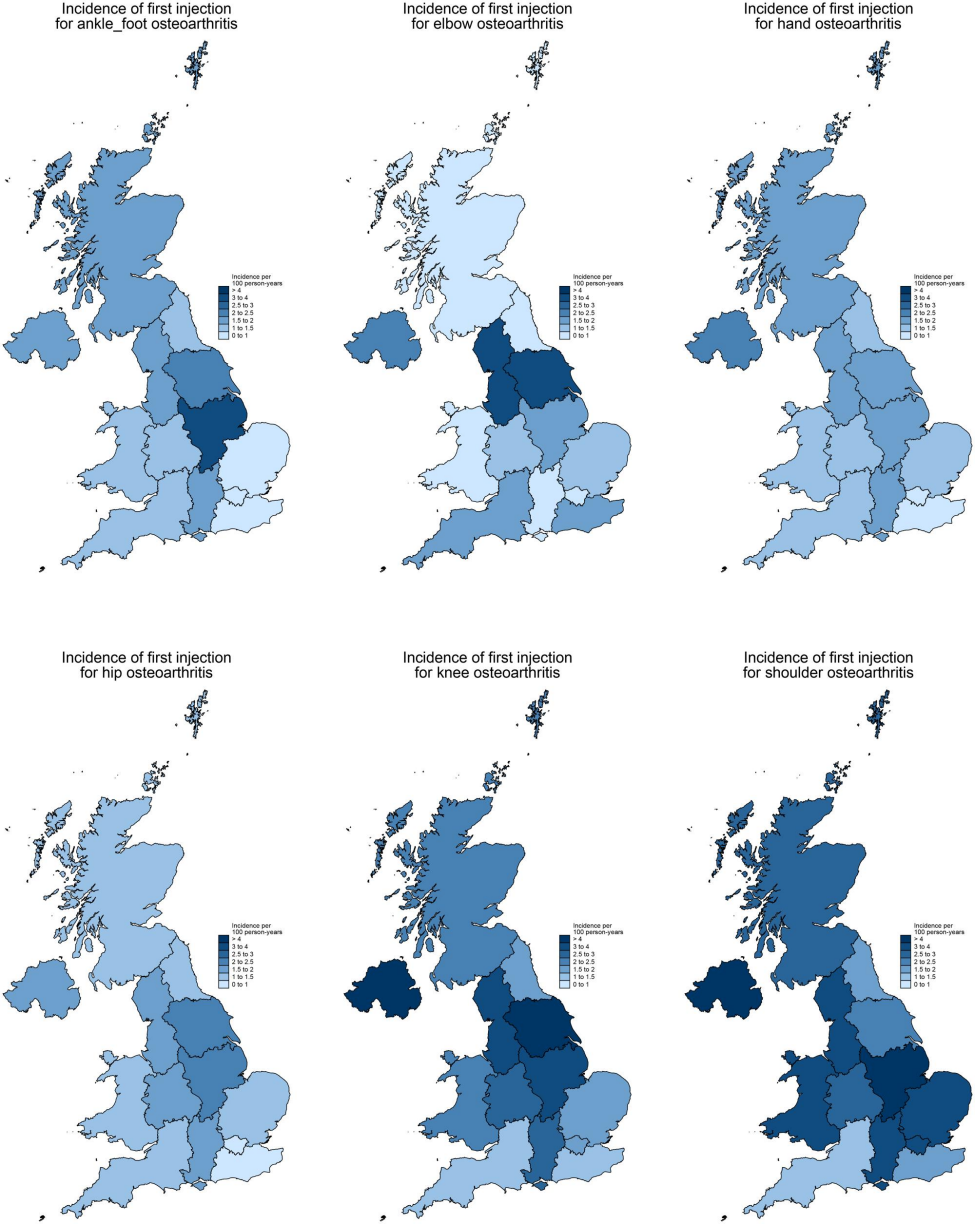
Incidence of first injection after OA diagnosis, by OA site and geographic region

Incidence rates of injections for all joints remained stable throughout the 2007–2019 period, with no clear upward or downward trend (Supplementary Fig. S1).

## Discussion

This large UK cohort of >220 000 patients with a first diagnosis of OA between 2005 and 2019 is consistent with clinical knowledge and previous literature, with the majority being female and diagnosed in their 60s [17]. Similarly, the most commonly affected OA anatomical sites were the knee (48.6%), hip (23.2%) and hand (12.6%), in alignment with previous studies [18]. In our cohort, 11% of participants received at least one IACI in primary care as treatment for OA, with multiple joints, the shoulder and the knee being the most frequently injected sites. This is consistent with the NICE guidelines of 2008 and 2014 that recommended IACIs be considered as an adjunct to core treatments for the relief of moderate to severe pain [9, 10].

Interestingly, the joints most commonly treated with IACIs in primary care differed from those most commonly affected by OA, potentially reflecting the influence of technical feasibility, perceived difficulty of the injection procedure and required resources in primary care settings [19, 20]. Among the patients who had a code specifying which joint was injected, the shoulder and knee joints were the most common individual joints injected, at almost 14% and 11% 5-year cumulative incidence, respectively. Although the shoulder joint was the most common individual joint to be injected, it was less commonly affected by OA than the knee, hip, hand and ankle or foot joints. Those with OA diagnoses in multiple joints had the highest overall incidence of joint injection, with 9% at 1 year and increasing to almost 16% at 5 years. Conversely, the elbow and hip joints were the least common individual joints injected, with a 5-year incidence of 5.2% and 5.6%, respectively.

Observed variance between affected and injected joints may be due to a combination of factors, including differences in disease progression and symptom severity. Perceived difficulties by the healthcare professional in primary care when injecting both the hip and hand joints may also be a factor, particularly for the hip joint, which requires image guidance, including fluoroscopy and ultrasound, that is not routinely available in primary care [20, 21]. Furthermore, the observed lower incidence of injection than expected for the joints most commonly affected by OA may reflect a higher incidence of injection of these joints being administered in secondary or private care. The NICE guidelines in 2008 and 2014 recommended advice on appropriate footwear for lower limb OA and to consider bracing, joint supports or insoles [9, 10]. The availability of non-pharmacological treatments for certain joints, which may have been preferred over an injection, could have influenced clinical decision-making. Additionally, NICE acknowledged that the majority of published evidence relates to OA of the knee [10]. This uneven evidence base may have influenced the perception of clinicians regarding the effectiveness of IACIs across different joints, and consequently prescribing behaviour in primary care.

Our analysis revealed substantial variation in the timing of IACIs in the natural history of OA across different joints. The most commonly injected sites, the knee and shoulder, had the lowest median years between OA diagnosis and first injection, with 0.7 and 0.3 median years, respectively. The elbow had the longest interval, at 2.1 years, followed by the hand and hip, both at a median of 1.5 years. In general, there was a trend of decreasing median time from diagnosis to injection for each subsequent injection. This suggests that once patients undergo their initial injection they tend to require subsequent injections at shorter intervals. This might imply progressive joint deterioration, a need for more frequent symptom management over time or decreasing effectiveness of subsequent injections.

Our analysis revealed consistently higher injection rates among women across all anatomical joint sites, with women receiving 0.5 more injections per 100 person-years for knee OA and 0.3 more for hip OA compared with men. This pattern likely reflects the higher overall burden of OA among women, as women have more severe disease with higher clinical pain and inflammation, which may drive a greater need for joint injections [22].

Regional variation in the incidence of IACIs in the UK was substantial, with Yorkshire and the Humber having the highest incidence for all joints combined at 3.8 injections per 100 patient-years, which is 3.2 times greater than the 1.2 injections per 100 patient-years seen in the South East Coast, the region with the lowest incidence. Incidence was highest for all joints combined among patients registered in GP practices in Yorkshire and the Humber, the East Midlands and Northern Ireland. Conversely, incidence was lowest in the South East Coast and South West. Factors contributing to this heterogeneity are likely to be multifaceted and include variability in healthcare services, interpretation of the evidence base and national guidance for IACIs, overlaid with regional clinical guidelines. Variation in the competency and confidence of individual GPs and physiotherapists to administer IACIs within primary care, is also likely to be a contributory factor [19]. Such variation raises important questions about equity of access to this treatment across the UK. However, the regional heterogeneity may also partly reflect differences in coding competency across GP practices and regions rather than solely representing true variation in clinical practice.

The stability in the incidence rates of injections for all joints throughout the 2007–2019 period suggests there were no major changes to practice on a national level. However, clinical practice may have changed considerably since the COVID-19 pandemic. NHS waiting lists for elective orthopaedic procedures were substantially impacted by the pandemic, and this may have amplified demand for IACIs in primary care given that qualitative evidence suggests that IACIs are offered to patients considering joint replacement surgery as a means of delaying or avoiding the procedure [19, 23]. Future research would be needed to determine whether this anticipated shift has occurred.

### Strengths and limitations

To our knowledge, this is the largest descriptive study of frequency and timing of IACI use for OA in the UK, encompassing 220 000 OA patients in a large national cohort representative of the general population according to age, sex and geographical region [14, 24], and benefitting from CPRD GOLD’s continuous data capture with an average follow-up of 13.5 years for enrolled patients. A systematic review of diagnostic coding validity in CPRD GOLD found that most diagnoses are well recorded [15]. Additionally, the diagnostic accuracy of coding of hip OA by GPs in CPRD GOLD has been assessed previously and was shown to have a high positive predictive value close to 80% [25]. However, equivalent validation data for other joints are not available. It is possible that the diagnostic accuracy of coding varies by joint and findings specific to individual joints should be interpreted with this caveat in mind.

Patients with a first diagnosis of OA between 1 January 2005 and 31 December 2019 were included, and data were extracted up until 28 August 2020. Thus patients diagnosed during the COVID-19 pandemic in the UK were not included and the majority of patients had years of follow-up. The decision to extract data up until 28 August 2020 was made because it was the start date of the project and to provide ≈8 months of follow-up for patients diagnosed in December 2019. The time from OA diagnosis to injection is unlikely to be materially affected by a less than full year of follow-up for these patients, as this measure is calculated only among patients who received both a diagnosis and an injection. A recognised limitation is that injection rates may be slightly underestimated for patients diagnosed in December 2019, as some who may have received an injection had follow-up continued beyond August 2020. This would have been a small number of patients and is unlikely to have influenced the overall findings.

More than 40% of IACI codes did not specify an anatomical location and thus we were unable to identify with certainty the injected joint. We sought to partially mitigate this by analysing patients with polyarthritis as a separate cohort. However, this remains an important limitation, as it is possible that the codes with missing information were not equally distributed across joints. One plausible explanation is that primary care clinicians may be more familiar with IACI codes for large joints such as the knee and shoulder than for smaller joints. Additionally, the IACI Read codes did not specify joint laterality, so we were unable to distinguish whether recurrent injections were performed at the same or opposite joint.

Additional limitations include that hospital-based or privately administered IACIs were not captured in our primary care–based study. The date of diagnosis recorded in the CPRD may not reliably reflect disease onset, which limits the accuracy of estimating IACI timing in the course of OA progression [25]. We were also unable to distinguish between primary and secondary OA.

## Conclusions

This large observational study of >220 000 UK patients with OA observed that 11% received at least one IACI in primary care on or after their diagnosis from 2005 to 2020. Where anatomical location was recorded (56%), the shoulder and knee were the most commonly injected joints. We identified substantial heterogeneity in the incidence of intra-articular injections in people with OA across different regions of the UK. Further research is required to understand the factors that may be contributing to the variation in time to injection and the geographical variation in IACI use to allow equitable treatment access for patients across the UK.

## Supplementary Material

rkag085_Supplementary_Data

## Data Availability

Data is available through application to CPRD and analytical code through request. Applications to access CPRD GOLD and linked data must be made directly to the CPRD in accordance with CPRD’s Research Data Governance process.
