# Supplementary material for: Characterising intra-articular corticosteroid injections use in UK primary care: a retrospective cohort study between 2005 and 2020
Source: Rheumatol Adv Pract. 2026 Jul 17;10(3):rkag085. doi: 10.1093/rap/rkag085 (PMC13472730; doi:10.1093/rap/rkag085)
Supplement: Supplementary file 1 [file rkag085_supplementary_data.docx]

# Supplementary Tables

***Supplementary Table S1: Read codes used to define osteoarthritis cohorts***

| **Joint** | **Read code** | **Read term** |
| --- | --- | --- |
| Ankle/foot | N052700 | Localised, secondary osteoarthritis of the ankle and foot |
|  | N05z712 | Foot osteoarthritis NOS |
|  | N053700 | Localised osteoarthritis, unspecified, of the ankle and foot |
|  | N05z713 | Toe osteoarthritis NOS |
|  | N05zS00 | Osteoarthritis NOS, of 1st MTP joint |
|  | N05zN00 | Osteoarthritis NOS, of ankle |
|  | N05zT00 | Osteoarthritis NOS, of lesser MTP joint |
|  | N05z700 | Osteoarthritis NOS, of ankle and foot |
|  | N051700 | Localised, primary osteoarthritis of the ankle and foot |
|  | N05zU00 | Osteoarthritis NOS, of IP joint of toe |
|  | N051E00 | Localised, primary osteoarthritis of toe |
|  | N05zP00 | Osteoarthritis NOS, of subtalar joint |
|  | N05z711 | Ankle osteoarthritis NOS |
|  | N05zR00 | Osteoarthritis NOS, of other tarsal joint |
|  | N05zQ00 | Osteoarthritis NOS, of talonavicular joint |
|  | N054700 | Oligoarticular osteoarthritis, unspecified, of ankle/foot |
|  | N05zM00 | Osteoarthritis NOS, of tibio-fibular joint |
| Elbow | N05z211 | Elbow osteoarthritis NOS |
|  | N051F00 | Localised, primary osteoarthritis of elbow |
|  | N05zC00 | Osteoarthritis NOS, of elbow |
|  | N052300 | Localised, secondary osteoarthritis of the forearm |
|  | N05z300 | Osteoarthritis NOS, of the forearm |
|  | N051300 | Localised, primary osteoarthritis of the forearm |
|  | N053300 | Localised osteoarthritis, unspecified, of the forearm |
|  | N052200 | Localised, secondary osteoarthritis of the upper arm |
|  | N051200 | Localised, primary osteoarthritis of the upper arm |
|  | N05z200 | Osteoarthritis NOS, of the upper arm |
|  | N053200 | Localised osteoarthritis, unspecified, of the upper arm |
| Hand | N052400 | Localised, secondary osteoarthritis of the hand |
|  | Nyu2A00 | [X]Other post-traumatic arthrosis/1st carpometacarpal joint |
|  | Nyu2B00 | [X]Other 2ndry arthrosis/1st carpometacarpal joints,bilaterl |
|  | N05z400 | Osteoarthritis NOS, of the hand |
|  | N05z412 | Thumb osteoarthritis NOS |
|  | N050111 | Heberdens' nodes |
|  | N05z411 | Finger osteoarthritis NOS |
|  | N05zF00 | Osteoarthritis NOS, of MCP joint |
|  | N05zH00 | Osteoarthritis NOS, of DIP joint of finger |
|  | N05zG00 | Osteoarthritis NOS, of PIP joint of finger |
|  | N053400 | Localised osteoarthritis, unspecified, of the hand |
|  | 2G26.00 | O/E - hands - Heberden's nodes |
|  | N053900 | Arthrosis of first carpometacarpal joint, unspecified |
|  | N051400 | Localised, primary osteoarthritis of the hand |
|  | N050700 | Heberden's nodes with arthropathy |
|  | N050112 | Bouchards' nodes |
|  | N051C00 | Primary arthrosis of first carpometacarpal joints, bilateral |
|  | N050100 | Generalised osteoarthritis of the hand |
|  | N050300 | Bouchard's nodes with arthropathy |
|  | N054400 | Oligoarticular osteoarthritis, unspecified, of hand |
|  | N052B00 | Post-traumatic arthrosis of first carpometacarpal jt bilat |
|  | Nyu2900 | [X]Other primary arthrosis of first carpometacarpal joint |
| Hip | N051A00 | Coxarthrosis resulting from dysplasia, bilateral |
|  | Nyu2200 | [X]Other dysplastic coxarthrosis |
|  | Nyu2E11 | [X] Unilateral secondary coxarthrosis |
|  | Nyu2400 | [X]Other secondary coxarthrosis, bilateral |
|  | Nyu2E00 | [X]Other secondary coxarthrosis |
|  | N052900 | Post-traumatic coxarthrosis, bilateral |
|  | Nyu2300 | [X]Other post-traumatic coxarthrosis |
|  | N053512 | Hip osteoarthitis NOS |
|  | N05z511 | Hip osteoarthritis NOS |
|  | N05zJ00 | Osteoarthritis NOS, of hip |
|  | Nyu2100 | [X]Other primary coxarthrosis |
|  | N051900 | Primary coxarthrosis, bilateral |
|  | N05zK00 | Osteoarthritis NOS, of sacro-iliac joint |
|  | N053511 | Otto's pelvis |
|  | N052500 | Localised, secondary osteoarthritis of pelvic region/thigh |
|  | N05z500 | Osteoarthritis NOS, pelvic region/thigh |
|  | N051500 | Localised, primary osteoarthritis of the pelvic region/thigh |
|  | N053500 | Localised osteoarthritis, unspecified, pelvic region/thigh |
| knee | Nyu2811 | [X] Unilateral secondary gonarthrosis |
|  | Nyu2800 | [X]Other secondary gonarthrosis |
|  | Nyu2700 | [X]Other secondary gonarthrosis, bilateral |
|  | N052A00 | Post-traumatic gonarthrosis, bilateral |
|  | N052C00 | Post-traumatic gonarthrosis, unilateral |
|  | N05z611 | Knee osteoarthritis NOS |
|  | N053611 | Patellofemoral osteoarthritis |
|  | N05zL00 | Osteoarthritis NOS, of knee |
|  | N072100 | Degenerative lesion of articular cartilage of knee |
|  | Nyu2511 | [X] Unilateral primary gonarthrosis |
|  | N051B00 | Primary gonarthrosis, bilateral |
|  | Nyu2500 | [X]Other primary gonarthrosis |
|  | N052600 | Localised, secondary osteoarthritis of the lower leg |
|  | N05z600 | Osteoarthritis NOS, of the lower leg |
|  | N051600 | Localised, primary osteoarthritis of the lower leg |
|  | N053600 | Localised osteoarthritis, unspecified, of the lower leg |
|  | N054600 | Oligoarticular osteoarthritis, unspecified, of lower leg |
|  | N05zM00 | Osteoarthritis NOS, of tibio-fibular joint |
| shoulder | N052100 | Localised, secondary osteoarthritis of the shoulder region |
|  | N05z100 | Osteoarthritis NOS, of shoulder region |
|  | N05z900 | Osteoarthritis NOS, of shoulder |
|  | N053100 | Localised osteoarthritis, unspecified, of shoulder region |
|  | N051100 | Localised, primary osteoarthritis of the shoulder region |
|  | N054100 | Oligoarticular osteoarthritis, unspecified, of shoulder |
|  | N052200 | Localised, secondary osteoarthritis of the upper arm |
|  | N051200 | Localised, primary osteoarthritis of the upper arm |
|  | N05z200 | Osteoarthritis NOS, of the upper arm |
|  | N053200 | Localised osteoarthritis, unspecified, of the upper arm |
| wrist | N052300 | Localised, secondary osteoarthritis of the forearm |
|  | N05z300 | Osteoarthritis NOS, of the forearm |
|  | N051300 | Localised, primary osteoarthritis of the forearm |
|  | N053300 | Localised osteoarthritis, unspecified, of the forearm |
|  | N05zE00 | Osteoarthritis NOS, of wrist |
|  | N05z311 | Wrist osteoarthritis NOS |
|  | N051D00 | Localised, primary osteoarthritis of the wrist |
|  | N05zD00 | Osteoarthritis NOS, of distal radio-ulnar joint |

***Supplementary Table S2: Read codes of injection site and osteoarthritis diagnosed joint***

| **Read Code Injection Site** | **Osteoarthritis diagnosed joint** | | | | | | | |  |
| --- | --- | --- | --- | --- | --- | --- | --- | --- | --- |
|  | **ankle_foot** | **elbow** | **hand** | **hip** | **knee** | **shoulder** | **wrist** | **multiple** | **Total** |
| **ankle_foot** | 213 | <5 | 23 | 41 | 120 | 9 | <5 | 135 | 545 |
| **elbow** | 7 | <5 | 37 | 42 | 95 | 6 | <5 | 35 | 230 |
| **hand** | 26 | <5 | 477 | 84 | 223 | 13 | 23 | 179 | 1,025 |
| **hip** | 22 | <5 | 73 | 1,306 | 338 | 14 | 10 | 375 | 2,140 |
| **knee** | 252 | 21 | 652 | 1,132 | 20,054 | 144 | 64 | 3,870 | 26,189 |
| **shoulder** | 430 | 31 | 1,535 | 2,476 | 5,341 | 1,387 | 139 | 2,224 | 13,563 |
| **wrist** | 12 | <5 | 127 | 59 | 112 | 8 | 87 | 119 | 527 |
| **Unspecific** | 1,096 | 93 | 4,060 | 5,622 | 18,124 | 993 | 327 | 4,531 | 34,846 |
| **Total** | 2,058 | 157 | 6,984 | 10,762 | 44,407 | 2,574 | 655 | 11,468 | 79,065 |

***Supplementary Table S3: Number of patients, percentage of women, and mean age at first osteoarthritis (OA) diagnosis with each combination of OA site***

| **Osteoarthritis site/s** | | | | **N** | **Women** | **Mean age at first OA diagnosis** |
| --- | --- | --- | --- | --- | --- | --- |
| knee |  |  |  | 106890 | 56.0% | 65.6 |
| hip |  |  |  | 50970 | 57.7% | 66.7 |
| hand |  |  |  | 27820 | 72.4% | 62.8 |
| ankle_foot |  |  |  | 8550 | 52.5% | 61.2 |
| shoulder |  |  |  | 5490 | 52.7% | 67.9 |
| wrist |  |  |  | 2351 | 59.4% | 66.0 |
| elbow |  |  |  | 824 | 30.0% | 61.8 |
| hip | knee |  |  | 6860 | 61.7% | 68.0 |
| hand | knee |  |  | 2969 | 69.8% | 64.7 |
| ankle_foot | knee |  |  | 1293 | 58.3% | 64.3 |
| hand | hip |  |  | 1137 | 73.6% | 65.6 |
| knee | shoulder |  |  | 971 | 59.4% | 69.0 |
| hip | shoulder |  |  | 464 | 61.6% | 69.6 |
| ankle_foot | hand |  |  | 424 | 72.9% | 61.7 |
| ankle_foot | hip |  |  | 405 | 59.0% | 64.7 |
| knee | wrist |  |  | 350 | 54.3% | 67.0 |
| hand | wrist |  |  | 225 | 66.7% | 66.0 |
| hand | shoulder |  |  | 196 | 71.9% | 67.8 |
| hip | wrist |  |  | 147 | 61.9% | 69.1 |
| elbow | knee |  |  | 112 | 36.6% | 62.6 |
| ankle_foot | shoulder |  |  | 81 | 55.6% | 66.9 |
| elbow | wrist |  |  | 73 | 54.8% | 59.7 |
| elbow | hip |  |  | 43 | 48.8% | 63.9 |
| ankle_foot | wrist |  |  | 40 | 55.0% | 67.1 |
| elbow | shoulder |  |  | 38 | 47.4% | 64.7 |
| elbow | hand |  |  | 30 | 36.7% | 61.0 |
| shoulder | wrist |  |  | 25 | 72.0% | 67.8 |
| ankle_foot | elbow |  |  | 10 | 20.0% | 58.4 |
| hand | hip | knee |  | 285 | 77.9% | 66.8 |
| hip | knee | shoulder |  | 194 | 64.9% | 69.9 |
| ankle_foot | hip | knee |  | 168 | 63.1% | 66.6 |
| ankle_foot | hand | knee |  | 110 | 67.3% | 63.4 |
| hand | knee | shoulder |  | 74 | 75.7% | 68.4 |
| hand | knee | wrist |  | 46 | 58.7% | 65.2 |
| ankle_foot | hand | hip |  | 43 | 67.4% | 64.8 |
| ankle_foot | knee | shoulder |  | 36 | 50.0% | 67.2 |
| hip | knee | wrist |  | 33 | 60.6% | 66.6 |
| hand | hip | shoulder |  | 28 | 64.3% | 67.8 |
| ankle_foot | hip | shoulder |  | 25 | 60.0% | 68.2 |
| hand | hip | wrist |  | 19 | 63.2% | 68.1 |
| ankle_foot | elbow | knee |  | 15 | 26.7% | 63.5 |
| ankle_foot | knee | wrist |  | 14 | 50.0% | 71.1 |
| elbow | knee | wrist |  | 14 | 78.6% | 64.1 |
| ankle_foot | hand | shoulder |  | 12 | 58.3% | 61.3 |
| elbow | hip | knee |  | 12 | 16.7% | 69.1 |
| ankle_foot | hand | wrist |  | 11 | 72.7% | 67.8 |
| knee | shoulder | wrist |  | 10 | 60.0% | 71.3 |
| ankle_foot | hand | hip | knee | 28 | 75.0% | 64.8 |
| ankle_foot | hip | knee | shoulder | 25 | 60.0% | 67.8 |
| hand | hip | knee | shoulder | 25 | 68.0% | 65.6 |

***Supplementary Figure S1: Yearly trends in the incidence of first injection after osteoarthritis (OA) diagnosis, by OA site***

*The incidence of first injection per 100 person-years is represented by a dot, with the line signifying the 95% confidence interval.*

*Alt text: Panel of eight charts showing yearly trends in the incidence of first injection after osteoarthritis diagnosis, by joint site, from 2007 to 2020.*
